# Bullous Pemphigoid Associated With COVID-19 Vaccines: An Italian Multicentre Study

**DOI:** 10.3389/fmed.2022.841506

**Published:** 2022-02-28

**Authors:** Carlo Alberto Maronese, Marzia Caproni, Chiara Moltrasio, Giovanni Genovese, Pamela Vezzoli, Paolo Sena, Giulia Previtali, Emanuele Cozzani, Giulia Gasparini, Aurora Parodi, Laura Atzori, Emiliano Antiga, Roberto Maglie, Francesco Moro, Elena Biancamaria Mariotti, Alberto Corrà, Sabatino Pallotta, Biagio Didona, Angelo Valerio Marzano, Giovanni Di Zenzo

**Affiliations:** ^1^Dermatology Unit, Fondazione Istituto di Ricovero e Cura a Carattere Scientifico Ca' Granda Ospedale Maggiore Policlinico, Milan, Italy; ^2^Department of Pathophysiology and Transplantation, Università degli Studi di Milano, Milan, Italy; ^3^Rare Diseases Unit, Section of Dermatology, Department of Health Sciences, Unità Sanitaria Locale Toscana Centro, European Reference Network-Skin Member, University of Florence, Florence, Italy; ^4^Department of Medical Surgical and Health Sciences, University of Trieste, Trieste, Italy; ^5^Dermatology Unit, Azienda Socio Sanitaria Territoriale Papa Giovanni XXIII Hospital, Bergamo, Italy; ^6^Clinical Chemistry Laboratory, Department of Clinical Pathology, Azienda Socio Sanitaria Territoriale Papa Giovanni XXIII Hospital, Bergamo, Italy; ^7^DiSSal, Dermatology Clinic, University of Genoa, San Martino Policlinic Hospital- Istituto di Ricovero e Cura a Carattere Scientifico, Genoa, Italy; ^8^Dermatology Clinic, Department Medical Sciences and Public Health, University of Cagliari, Cagliari, Italy; ^9^Department of Health Sciences, Section of Dermatology, University of Florence, Florence, Italy; ^10^Molecular and Cell Biology Laboratory, Istituto Dermopatico dell'Immacolata - Istituto di Ricovero e Cura a Carattere Scientifico, Rome, Italy; ^11^Dermatology Clinic, Istituto Dermopatico dell'Immacolata - Istituto di Ricovero e Cura a Carattere Scientifico, Rome, Italy; ^12^Rare Disease Unit, Istituto Dermopatico dell'Immacolata - Istituto di Ricovero e Cura a Carattere Scientifico, Rome, Italy

**Keywords:** bullous pemphigoid, vaccine, COVID-19, autoantibodies, SARS-CoV-2, triggering factors, BP180, BP230

## Abstract

Bullous pemphigoid (BP) is an autoimmune bullous disease caused by circulating autoantibodies toward the hemidesmosomal antigens BP180 and BP230. Cases of BP have been described following vaccinations against tetanus, poliomyelitis, diphtheria, influenza, pneumococcus, meningococcus, hepatitis B and rabies. The putative mechanism by which COVID-19-vaccines may induce BP has not been clarified. An Italian multicentre study was conducted to collect clinical, histopathological and immunopathological data of patients with BP associated with COVID-19-vaccines. Twenty-one cases were collected, including 9 females and 12 males (M/F = 1.3) with a median age at diagnosis of 82 years. Seventeen patients received the COMIRNATY Pfizer-BioNTech vaccine, two the Moderna mRNA-1273 vaccine, one the ChAdOx1/nCoV-19-AstraZeneca/ Vaxzevria vaccine and one received the first dose with the ChAdOx1/nCoV-19-AstraZeneca/Vaxzevria vaccine and the second dose with the COMIRNATY Pfizer-BioNTech vaccine. Median latency time between the first dose of anti-SARS-CoV-2 vaccine and the onset of cutaneous manifestations was 27 days. Median BPDAI at onset was 42. Eleven out of seventeen patients (65%) had positive titres for anti-BP180 antibodies with a median value of 106.3 U/mL on ELISA; in contrast, only five out of seventeen (29%) were positive for anti-BP230 antibodies, with a median of 35.3 U/mL. In conclusion, in terms of mean age, disease severity at diagnosis and clinical phenotype vaccine-associated BP patients seem to be similar to idiopathic BP with an overall benign course with appropriate treatment. On the other hand, the slight male predominance and the reduced humoral response to BP230 represent peculiar features of this subset of patients.

## Introduction

Bullous pemphigoid (BP) is an autoimmune bullous disease caused by circulating autoantibodies toward the hemidesmosomal antigens BP180 and BP230 (1).

Although the majority of cases are considered idiopathic, several trigger factors have been described in literature, such as UV light, radiation, drugs and trauma. Moreover, cases of BP developed following vaccine injection have recently been reported, with a variable latency time, mostly <1 month (2–5). Specifically, multiple vaccinations are reported as trigger for BP, including the ones for influenza (4, 6), pneumococcus (7), meningococcus (2, 8), varicella-zoster (3), rabies (9) and hexavalent (diphtheria, tetanus, pertussis, poliomyelitis, hepatitis B, and Haemophilus influenzae B) (2, 10).

More recently, both new onset and reactivation of BP have been observed after the inoculation of SARS-CoV-2 vaccines (11–14). The putative mechanism by which COVID-19 vaccines may induce BP has not been thoroughly investigated.

Autoimmune mechanisms following SARS-CoV-2 infection may be associated with molecular mimicry (15, 16). On the other hand, vaccination may activate B and T-cell immunity, triggering an autoimmune response in genetically predisposed individuals (17).

The present multicentre study aimed at investigating the demographics, clinical and immunopathological features of SARS-CoV-2 vaccine-associated BP.

## Methods

SARS-CoV-2 vaccine-associated BP patients examined between February 1, 2021, and November 15, 2021, were included in the present multicentre study involving six Dermatology Clinics (Milan, Cagliari, Florence, Genoa, Bergamo and Rome). The following eligibility criteria were adopted: (1) age of 18 years or older; (2) recent anti-SARS-CoV2 vaccination (<2 months after either the I or II dose); (3) a Naranjo score of 4 or above concerning the association between BP and SARS-CoV-2 vaccine; (4) absence of newly prescribed medications (in the 3 months preceding BP onset) or dipeptidyl peptidase 4 inhibitors; (5) diagnosis of BP based on typical findings on clinical, histopathological and/or immunopathological [IgG and/or C3 deposits along the dermal-epidermal junction (DEJ) on direct immunofluorescence (DIF) and/or indirect immunofluorescence (IIF) microscopy] examinations. The study was conducted in accordance with the Declaration of Helsinki guidelines and all patients gave written informed consent. The present study is a combined retrospective and prospective study. Clinical data were collected from electronic charts but also directly from patients at baseline or during the follow up visit. Skin manifestations were directly evaluated by a dermatologist. Each patient was examined at least twice (during the period of skin manifestations and after 3 months). Response to treatment was evaluated according to the recommendations from the International Pemphigoid Committee (18). Each participating center was asked to provide the following data: sex; age at onset; SARS-CoV-2 vaccine type; first and second dose date; time from SARS-CoV2 vaccine administration and BP onset; Naranjo score; comorbidities and concomitant medications; clinical scores [Autoimmune Bullous Skin Disorder Intensity Score (ABSIS) and Bullous Pemphigoid Disease Area Index (BPDAI), histopathological and immunopathological features (direct and/or indirect immunofluorescence, ELISA-tests); COVID-19 medications and duration of follow-up.

To identify anti-BP180 and anti-BP230 autoantibodies in patients' serum, commercial ELISA kits (Euroimmun, Padova, Italia) were used, in accordance with the manufacturer's instructions. A cut-off value of >20 U/mL was used for both type of test. As for DIF microscopy the sections stained with fluorescein isothiocyanate-conjugated goat anti-human Ig and C3 (Kallestad Diagnostic, Chaska, MN, USA), were analyzed under a fluorescence microscope. DIF results were recorded by taking into consideration the nature of the immune deposits (IgG, IgA, IgM, C3), the location of the immune deposits and the extent and the pattern of immune complex deposits (granular or linear). IIF was performed on slides containing human epithelial substrate and human salt-split skin as described (19).

## Results

**Figure 1:**
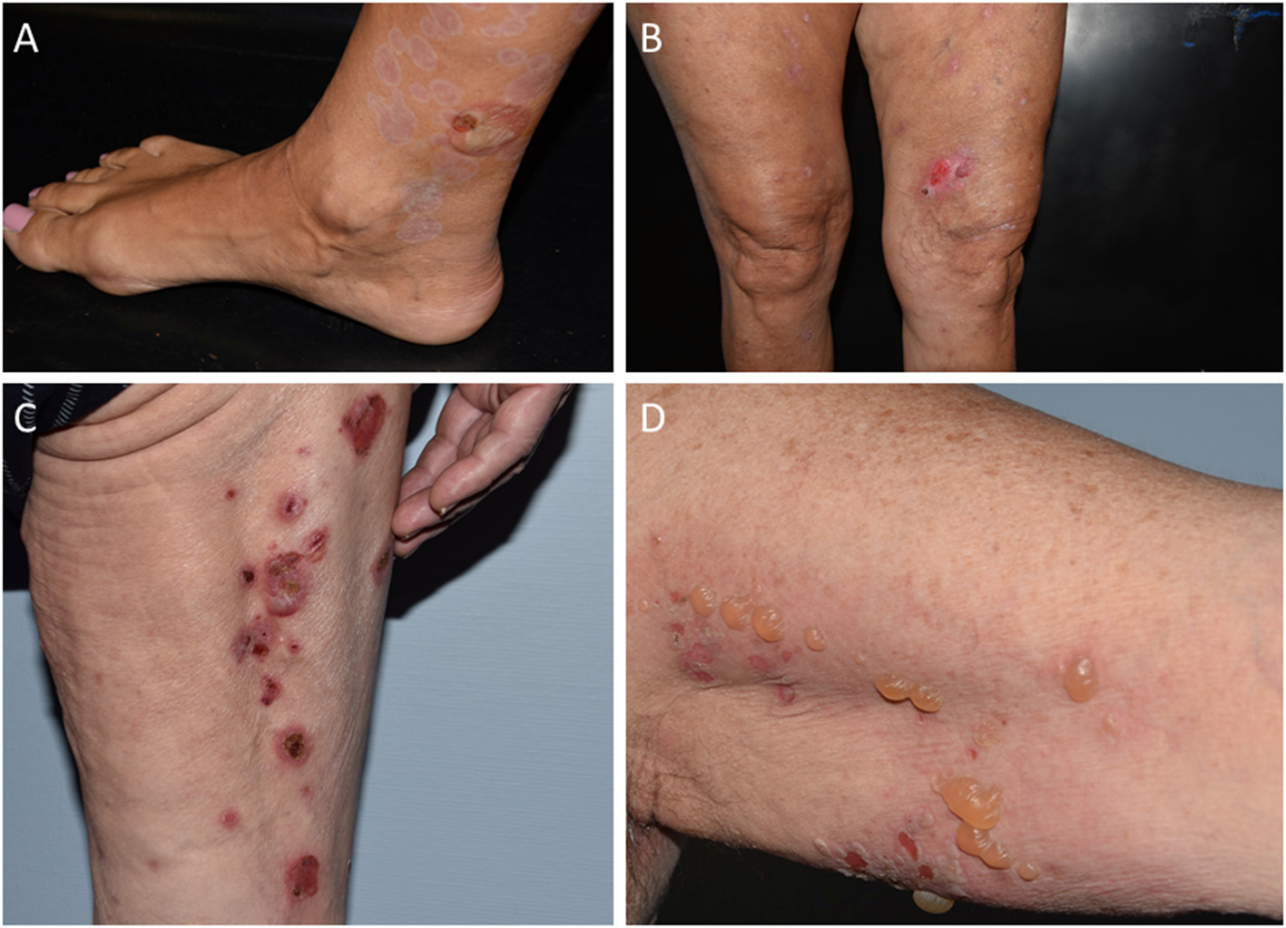
Clinical spectrum of vaccine-associated BP patients. **(A)** Acral distribution of active blister associated with older lesions in partial resolution, resulting in mild erythema and hypopigmentation. **(B)** Sero-hemorrhagic bullous, pruritic eruption on medial surface of left thigh, surrounded by multiple prurigo-like specific lesions. **(C)** Linear distribution of erythematous blisters, resulting in crusts and erosions. **(D)** Blisters and erosions with mild erythema located on left axilla.

**Figure 2:**
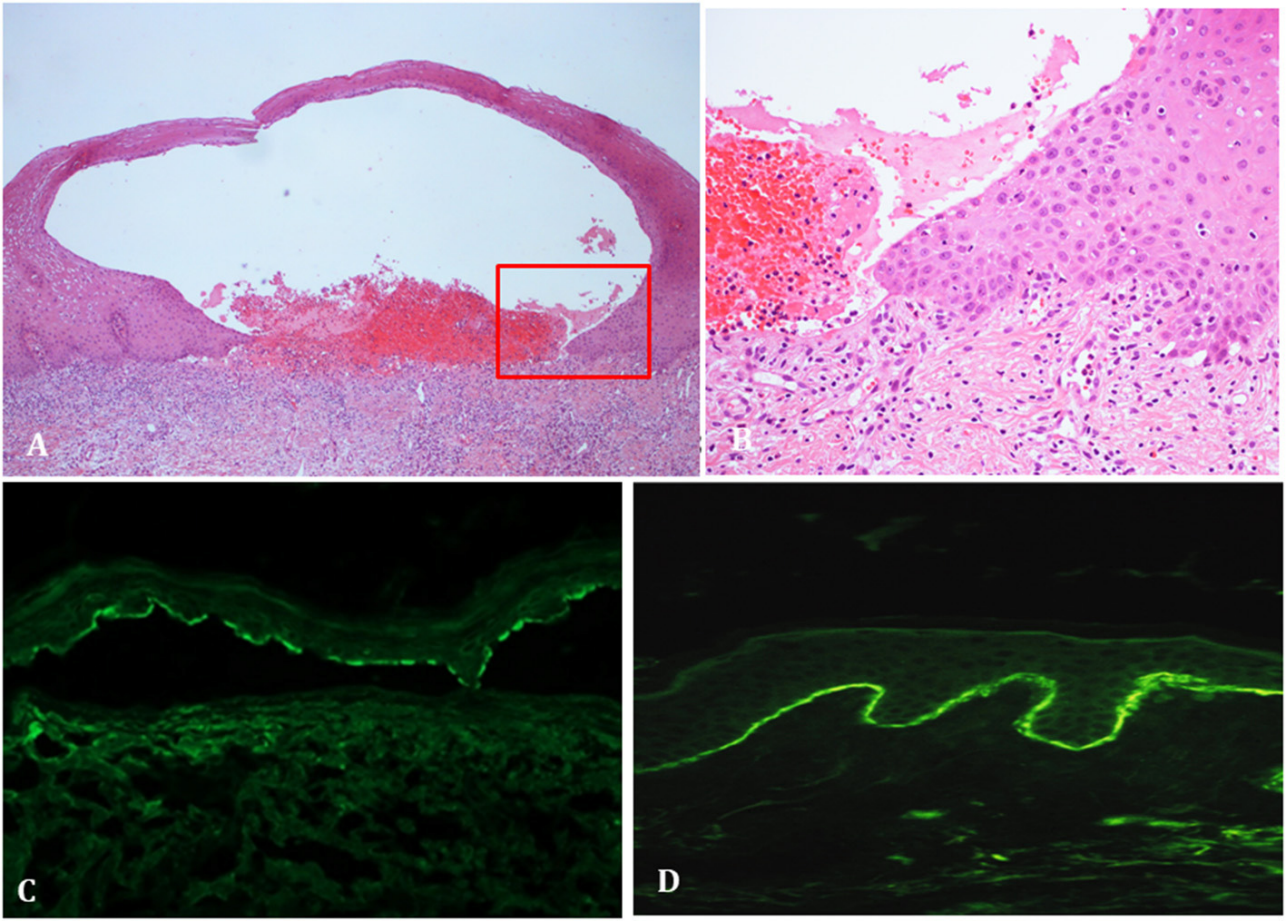
Histopathological and immunopathological findings of vaccine-associated BP patients. **(A)** Histopathology showing subepidermal detachment accompanied by inflammatory infiltrates in the dermis (hematoxylin and eosin staining). **(B)** Close-up view revealing the supepidermal detachment with a dermal inflammatory infiltrate, mainly consisting of lymphocytes and eosinophils (hematoxylin and eosin staining). **(C)** Salt splin skin in indirect immunofluorescence shows IgG deposits along the dermo-epidermal junction. **(D)** Direct immunofluorescence shows linear IgG/C3 deposits along the dermo-epidermal junction.

Twenty-one cases of SARS-CoV2 vaccine-associated BP were collected, including 9 females and 12 males (M/F = 1.3) with a median age at diagnosis of 82 (IQR: 74–85.5) years (Table 1). Seventeen patients received the COMIRNATY Pfizer-BioNTech vaccine, two the Moderna mRNA-1273 vaccine, one the ChAdOx1/nCoV-19-AstraZeneca/ Vaxzevria vaccine and one received the first dose with the ChAdOx1/nCoV-19-AstraZeneca/Vaxzevria vaccine and the second dose with the COMIRNATY Pfizer-BioNTech vaccine. Median latency time between the first dose of SARS-CoV2 vaccine and the onset of cutaneous manifestations was 27 (IQR: 7–34) days (Table 1). The onset of clinical manifestations occurred in eight patients after the first dose and in 13 after the second dose. Among those with BP appearance between the first and the second dose, median latency time was 6.5 (IQR: 4–7) days from the first dose, whereas among those with BP onset after the second dose, the median latency was 7 (IQR: 4–14.5) days from the second dose [and 32 (IQR: 27–36.5) days from the first one]. Nineteen patients had a Naranjo score ≥6 while two had a Naranjo score of 4. Baseline BPDAI scores were available for all patients. Median BPDAI at onset was 42 (IQR: 18.5–61). Baseline ABSIS scores were available for 16 out of 21 patients. Median ABSIS at onset was 30 (IQR: 15.75–58.5) (Table 1). Laboratory exams were within normal ranges. Eleven out of seventeen patients (64.7%) had positive (>20 U/mL) titres for anti-BP180 antibodies with a median value of 106.3 U/mL on ELISA (IQR: 40–237.5 U/mL); in contrast, only 5 out of 17 (29.4%) were positive for anti-BP230 antibodies, with a median of 35.3 U/mL on ELISA (IQR: 25.9–249.3 U/mL) (Table 2). The clinicopathological picture was typical across our cohort (Figures 1, 2). DIF showed linear IgG and C3 deposits along the DEJ (9 out of 18 cases), isolated linear C3 deposits along the DEJ (6/18), isolated linear IgG deposits along the DEJ (1/18), isolated granular C3 deposits along the DEJ (1/18). DIF turned out negative in one case. IIF performed on salt-split human skin revealed epidermal side binding in all tested cases (13/21) (Table 2).

**Table 1:** Demographics and clinical features of reported cases.

**N**.	**Sex, age (years)**	**Vaccine**	**Concomitant medications**	**Latency from the 1^st^ dose (days)**	**Naranjo score^#^**	**Baseline BPDAI**	**Baseline ABSIS**	**Treatment**	**BPDAI at 3 months**	**ABSIS at 3 months**
1	F, 84	Pfizer	Alendronate	25	6	70	21	Topical and systemic CS plus doxycycline	0	0
2	M, 83	Pfizer	Allopurinol, amiodarone, amlodipine, bicalutamide, clonidine, furosemide, insulin, valsartan, warfarin	32	6	50	18	Topical and systemic CS plus doxycycline	0	0
3	F, 56	Moderna	none	7	6	17	4.5	Topical CS plus doxycycline	0	0
4	M, 79	Pfizer	ASA, amiodarone, atorvastatin, clopidogrel, hydrochlorothiazide, olmesartan, pantoprazole, tamsulosin	4	6	23	10	Topical CS plus doxycycline	0	0
5	M, 86	Pfizer	Amiodarone, atorvastatin, clopidrogrel, domperidone, escitalopram, hydrochlorothiazide, levodopa/benserazide	37	6	20	12	Topical CS	0	0
6	M, 91	Pfizer	Allopurinol, atorvastatin, furosemide, insulin, nebivolol	28	6	80	30	Topical and systemic CS	0	0
7	M, 86	Pfizer	ASA, fenofibrate, isosorbide, ivabradine, pyridostigmine	36	6	52	20	Topical and systemic CS plus doxycycline	0	0
8	F, 84	Moderna	Amlodipine, glimepiride, metformin, levothyroxine	7	6	40	15	Topical and systemic CS plus doxycycline	0	0
9	M, 84	Pfizer	None	23	6	37	54	Systemic CS	0	0
10	F, 82	Pfizer	None	34	6	52	90	Systemic CS	6	27
11	M, 76	Pfizer	Candesartan, hydrochlorothiazide	34	6	47	70	Systemic CS	NA	NA
12	M, 78	Pfizer	none	4	4	42	NA	Topical CS	0	NA
13	F, 90	Pfizer	Allopurinol, hydrochlorothiazide, losartan	28	4	142	NA	Topical and systemic CS	25	NA
14	M, 90	Pfizer	Alfuzosin, allopurinol, darbepoetin alfa, furosemide, levothyroxine, pregabalin, warfarin	64	6	20	NA	Systemic CS	0	NA
15	M, 72	Pfizer	Insulin, telmisartan	16	6	80	NA	Topical and systemic CS plus MTX	29	NA
16	M, 80	Pfizer	ASA, amlodipine, atenolol, atorvastatin, finasteride, salmeterol/fluticasone, zofenopril	6	6	71	90	Topical and systemic CS	51	70
17	F, 77	AstraZeneca	Amlodipine, bisoprolol, furosemide, ramipril, sertraline	3	8	42	60	MTX	0	0
18	F, 60	Pfizer	None	75	6	10	36	Systemic CS	0	0
19	F, 70	Pfizer	None	27	6	15	35	Systemic CS	1	5
20	F, 72	AstraZeneca (1^st^ dose), Pfizer (2^nd^ dose)	ASA, amlodipine, levothyroxine, perindopril, simvastatin	7	6	15	NA	Systemic CS plus dapsone	3	NA
21	M, 85	Pfizer	ASA, atenolol, dutasteride, indapamide, perindopril, tamsulosin	27	6	15	30	Systemic CS	41	50

# *Naranjo scale interpretation: doubtful (≤0), possible (1-4), probable (5-8), definite (≥9)*.

*CS, corticosteroids; MTX, methotrexate; NA, not available; ABSIS, Autoimmune Bullous Skin Disorder Intensity Score; ASA, acetylsalicylic acid; BPDAI, Bullous Pemphigoid Disease Area Index*.

**Table 2:** Immunopathological features of reported cases.

**N**.	**Histopathology^§^**	**DIF**	**IIF**	**ELISA IgG anti-BP180 (U/mL)**	**ELISA IgG anti-BP230 (U/mL)**
1	+	Linear IgG/C3 deposits along the DEJ	IgG along the DEJ. SSS: roof	40	8.5
2	+	Linear IgG/C3 deposits along the DEJ	IgG along the DEJ. SSS: roof	492.1	425
3	+	Neg	IgG along the DEJ. SSS: roof	136.8	73.6
4	+	Linear IgG/C3 deposits along the DEJ	IgG along the DEJ. SSS: roof	237.5	0
5	+	Linear IgG/C3 deposits along the DEJ	IgG along the DEJ. SSS: roof	46.9	9.7
6	+	Linear IgG/C3 deposits along the DEJ	IgG along the DEJ. SSS: roof	14.9	0
7	+	Linear IgG/C3 deposits along the DEJ	NA	NA	NA
8	+	Linear IgG/C3 deposits along the DEJ	IgG along the DEJ. SSS: roof	247.2	5.7
9	+	Linear C3 deposits along the DEJ	NA	0	0
10	+	Linear IgG/C3 deposits along the DEJ	NA	0	0
11	+	Linear C3 deposits along the DEJ	NA	0	0
12	+	NA	IgG along the DEJ. SSS: roof	29.1	29.6
13	+	Linear IgG deposits along the DEJ	IgG along the DEJ. SSS: roof	106.3	2.9
14	+	Linear C3 deposits along the DEJ	neg	3.3	1.3
15	+	Linear C3 deposits along the DEJ	neg	140.4	0
16	+	Linear IgG/C3 deposits along the DEJ	IgG along the DEJ. SSS: roof	NA	NA
17	+	Linear C3 deposits along the DEJ	IgG along the DEJ. SSS: roof	52.9	22.2
18	+	Granular C3 deposits along the DEJ	IgG along the DEJ. SSS: roof	23.7	35.3
19	+	Linear C3 deposits along the DEJ	IgG along the DEJ. SSS: roof	5.5	1.8
20	+	NA	NA	NA	NA
21	+	NA	NA	NA	NA

§ *Consistent with bullous pemphigoid, i.e., subepidermal blistering and eosinophil-rich infiltrates*.

*DIF, direct immunofluorescence; IIF, indirect immunofluorescence; DEJ, dermal-epidermal junction; SSS, salt-split skin; NA, not available*.

Treatment included systemic corticosteroids (7), topical and systemic corticosteroids (3), topical and systemic corticosteroids plus doxycycline (4), topical corticosteroids plus doxycycline (2), topical and systemic corticosteroids plus methotrexate (1), systemic corticosteroids plus dapsone (1) and topical corticosteroids alone (2), methotrexate alone (1) (Table 1).

At 3 months, 13 patients achieved a complete response, whereas 6 had a partial response and one had stable disease [mean ABSIS percentage change = −80.75% (SD ± 44.25; *n* = 15); mean BPDAI percentage change = −78.14% (SD ± 60.21; *n* = 20)] (Table 1).

## Discussion

Vaccination has rarely been associated with new-onset dermatoses as well as flaring of pre-existent dermatological disease (11). SARS-CoV-2-vaccine-associated cutaneous eruptions encompass a growing spectrum of clinicopathological varieties, including local injection site reactions, urticarial eruptions, morbilliform eruptions, pernio/chilblain-like lesions, cosmetic filler reactions, herpes zoster and herpes simplex flares, pityriasis rosea-like eruptions (11, 20, 21). Autoimmune bullous skin diseases have also been observed following SARS-CoV-2-vaccination, with approximately 34 individual cases of vaccine-associated BP currently described (12, 14, 17, 22–28) (Supplementary Table 1). According to the registry-based studies by McMahon et al., BP-like eruptions accounted for 20% (12/58) of biopsy-proven SARS-CoV-2-vaccine-associated cutaneous reactions and 1.5% overall (11, 22).

The present multicentre study reports 21 cases of SARS-CoV-2 vaccine-associated BP, representing the largest case series to date.

Median age at onset (81 years) was in line with published observations [82.5 (IQR: 71.25–84.75) years; *n* = 24/34 with age available] (23–28). Likewise, sex distribution showed a slight male sex preference in both our cohort (M:F = 1.3) and available reports (M:F = 1.2; *n* = 22 with gender available) (23–28).

Vaccine-induced BP was more frequently associated with the Pfizer vaccine (80.1 vs. 67.6% of available reports), as compared with other mRNA- (Moderna mRNA-1273, 9.5 vs. 29.4% of available reports) or vector-based vaccines (ChAdOx1/nCoV-19-AstraZeneca/Vaxzevria, 9.5 vs. 2.9% of available reports). In line with our data McMahon and coworkers have recently found more BP cases associated with Pfizer vaccine than with Moderna (64 vs. 36%) (21). It is unclear whether this association depends on the greater employment of the Pfizer vaccine or if it underlies a deeper pathogenetic link. In fact, at the time of this study the percentage of Pfizer administration to adult patients was much higher (69.4%) in comparison with Moderna (18.3%), AstraZeneca (10.6%) and Janssen (1.7%) (29). In addition, in the present and all reported studies the sample size is too small to get meaningful result in term of association with a specific vaccine. To assess a possible link further studies with a large sample size standardized by specific vaccine administration should be performed.

Overall, the median latency time between the first dose and onset of cutaneous lesions was 27 days, which is notably higher than that of available reports [median latency time from the first dose to onset: 7 (IQR: 4–22.5) days, *n* = 17 with timing data available]. However, direct comparison with published cases is hindered by the lack of precise reporting of vaccination timings—especially in the case of vaccines with longer, variable time intervals between doses (e.g., Moderna mRNA-1273 vaccine, ChAdOx1/nCoV-19-AstraZeneca/Vaxzevria). Latency time from last dose was the preferred way of reporting across the literature. In our study, among those with BP appearance between the first and the second dose (*n* = 8), the median latency time was 6.5 (IQR: 4–7) days after the first dose, in line with available reports [median = 6 (IQR: 3–7.75) days, *n* = 12]. Similarly, those with BP onset after the second dose (*n* = 13) had a median latency time of 7 (IQR: 4–14.5) days from the latter, which is in agreement with the literature [median = 7 (2.5–14) days, *n* = 9]. Speculatively, a latency time shorter than a week (i.e., the minimum time required for antibody production) since the first dose may hint at a role for the stimulation of pre-existent autoimmunity in the pathogenesis of SARS-CoV-2-vaccine-associated BP. Conversely, late onset SARS-CoV-2-vaccine-associated BP may result from a dysregulated primary immune response triggered by the vaccine. Of note, it has been suggested that a one-month latency period from the time of vaccination may be appropriate for anti-basement membrane antibody induction (30).

Clinically, the presentation of SARS-CoV-2-vaccine-associated BP appears to be typical with tense bullae on an erythematous base, various degrees of cutaneous involvement, and an overall benign course with appropriate treatment (only patient n. 21 had stable disease at 3 months). Although many published reports describe a similarly favorable course (17, 24–28), in the study by Tomayko *et al*., five patients had ongoing disease after a follow-up period ranging from 23 to 105 days (12). Our sample size prevents the possibility to reliably compare different treatments. However, most of the subjects were easily controlled with treatment regimens concepted for milder forms of BP (i.e., topical steroids, low-to-moderate doses of systemic corticosteroids, doxycycline), supporting the assumption that the majority of COVID-19 induced BP cases would be non-severe (17, 24–26). Systemic corticosteroids as well as immunosuppressive adjuvants required to achieve disease control in BP may affect the efficacy of anti-SARS-CoV-2 vaccines. Humoral and cellular immune responses to COVID-19 mRNA vaccines are reduced in patients with immune-mediated inflammatory diseases on background methotrexate (31). Moreover, treatment with mycophenolate mofetil and rituximab also compromise anti-SARS-CoV-2 antibody responses (32). However, according to the updated international recommendations for the management of autoimmune bullous diseases during COVID-19 pandemic, lowering the dosage of immunomodulatory medications before or during the vaccination is not advisable due to the risk of exacerbations (33).

Immunopathological findings also seem to be typical, highlighting linear IgG/C3 deposits along the DEJ on DIF and epidermal side binding on SSS IIF in the vast majority of cases. The serological landscape of SARS-CoV2 vaccine-associated BP is dominated by the presence of anti-BP180 autoantibodies with a frequency (65%) comparable with literature data (34, 35). Of note, positivity for anti-BP230 autoantibodies was infrequent in our cohort with a frequency of reactivity (29%) sharply lower than that previously reported (34, 35). Previous studies, investigating the dynamics of immune response to BP antigens, described that it involves at first extracellular antigens/epitopes (BP180-NC16A domain) followed by intracellular ones (BP230) possibly exposed after tissue damage (36, 37). In the light of these findings, it could be speculated that in vaccine-associated BP, due to very short disease duration, the induction of secondary response to BP230 is not always detectable.

Vaccine-induced BP could stem from vaccine-mediated stimulation of pre-existent, sub-clinical autoreactivity against hemidesmosomal components, as seen in a proportion of pruritic dermatoses of the elderly characterized by IgG-mediated autoimmunity against BP230 (38). However, limited anti-BP230 reactivity across our cohort and published reports would not encourage this interpretation. SARS-CoV-2 vaccine-associated BP may be driven by a specific pathogenetic process in genetically predisposed individuals. Prior to translation, mRNA vaccines could trigger several pro-inflammatory pathways via Toll-like receptor (TLR)-3, TLR7 and TLR8 binding (39). Moreover, through cytokine modulation, novel antigens and adjuvants could promote T-cell-dependent immune responses leading to the production of self-reactive B cells. Indeed, SARS-CoV-2-reactive T cell clones have been reported in the infiltrate of two elderly men with vaccination-induced BP (17). A contributing role of hollow needle-induced tissue disruption during vaccination has also been hypothesized (14, 40). Although no new medications were introduced in our cohort in the 3 months preceding BP onset, the majority of our patients was receiving polypharmacy for various indications. Indeed, drugs potentially linked to drug-induced BP, including antihypertensives, salicylates and diuretics, had been administered for years in some of our cases (Table 1). It is not unconceivable that anti-SARS-CoV-2 vaccines may have created a suitable immune environment to make these individuals more prone to drug-induced BP (41).

In conclusion, SARS-CoV-2-vaccine-associated BP seems to be superimposable to idiopathic BP in terms of median age at onset and clinical presentation. On the other hand, slight male predominance and reduced humoral response to BP230 could represent peculiar features of this subset of patients. A close relationship between vaccination and BP onset is difficult to prove considering the extensive vaccination of the adult population during COVID-19 pandemic. However, the recent immunopathological findings by Gambichler *et al*. (17) as well as timing reported across our cohort and published cases support the hypothesis of a causal link between SARS-CoV-2 vaccine and BP development. Further research is warranted to better define the nature of SARS-CoV-2-vaccine-associated immune dysregulation leading to BP.

## Data Availability Statement

The raw data supporting the conclusions of this article will be made available by the authors, without undue reservation.

## Ethics Statement

The studies involving human participants were reviewed and approved by Istituto Dermopatico dell'Immacolata (IDI)-IRCCS. The patients/participants provided their written informed consent to participate in this study.

## Author Contributions

MC, GD, and AM: designed the study. CAM GGe, PV, PS, EC, GGa, AP, EA, LA, RM, MC, EM, AC, SP, BD, and AM: enrolled patients. FM, RM, and GP: carried out the experiment. CAM, CM, GG, MC, GD, and AM: wrote the manuscript. CAM, MC, and GD: contributed to the interpretation of the results. GD and AM: conceived and planned the experiments. All authors contributed to the article and approved the submitted version.

## Funding

This study was supported by the Progetto Ricerca Corrente and Ricerca Finalizzata N 12367807 of the Italian Ministry of Health, Rome, Italy.

## Conflict of Interest

The authors declare that the research was conducted in the absence of any commercial or financial relationships that could be construed as a potential conflict of interest.

## Publisher's Note

All claims expressed in this article are solely those of the authors and do not necessarily represent those of their affiliated organizations, or those of the publisher, the editors and the reviewers. Any product that may be evaluated in this article, or claim that may be made by its manufacturer, is not guaranteed or endorsed by the publisher.
