# Supplementary material for: Bullous Pemphigoid Associated With COVID-19 Vaccines: An Italian Multicentre Study
Source: Front Med (Lausanne). 2022 Feb 28;9:841506. doi: 10.3389/fmed.2022.841506 (PMC8918943; doi:10.3389/fmed.2022.841506)
Supplement: Supplementary file 1 [file Table_1.docx]

**Table 1 supplementary. Clinical and immunopathological features of published cases.**

| **Ref.** | **Sex, age (years)** | **Vaccine** | **Timing** | **DIF** | **IIF** | **ELISA**  **anti-BP180 (U/mL)** | **ELISA**  **anti-BP230 (U/mL)** | **Naranjo score** |
| --- | --- | --- | --- | --- | --- | --- | --- | --- |
| Pérez-López | F, 78 | Pfizer | 3 days after the first dose | + along the DEJ. | + | NA | NA | 8 |
| Dell’Antonia | M, 83 | Pfizer | 7 days after the first dose | C3+ along the DEJ. | NA | NA | NA | 9 |
| Agharbi | M, 77 | AstraZeneca | 1 day after the first dose | IgG+ along the DEJ. | + along the DEJ. | NA | NA | NA |
| Young | M, 68 | Pfizer | 3 days after the first dose | IgG+/C3+ along the DEJ. | - | NA | NA | NA |
| Schmidt | F, 84 | Moderna | “Few” days after the first dose | NA | anti-BP180=1:320/  anti-BP230+++ | NA | NA | NA |
| Nakamura | F, 83 | Pfizer | 3 days after the second dose | NA | NA | 11530 | NA | NA |
| Tomayko | F, 97 | Pfizer | 2 days after the second dose | IgG+, C3+, IgA+ along the DEJ. | IIF (SSS): roof | 130 | 81 | NA |
|  | M, 75 | Pfizer | 10 days after the second dose | C3+ along the DEJ. | + | 169 | NA | NA |
|  | M, 64 | Pfizer | 14 days after the second dose | C3+ along the DEJ | IIF (SSS): floor | 26 | 82 | NA |
|  | M, 82 | Pfizer | 1 days after the second dose | IgG+, C3+, weak IgA+ along the DEJ | IIF (SSS): roof | neg | neg | NA |
|  | F, 95 | Pfizer | 5 days after the first dose | IgG+, C3+, weak IgA+ along the DEJ | IIF (SSS): roof | neg | neg | NA |
|  | M, 87 | Moderna | 21 days after the second dose | C3+ along the DEJ | anti-BP180+/anti-BP230+ | NA | NA | NA |
|  | F, 42 | Moderna | 3 days after the second dose | IgG+, C3+, weak granular IgM+ | + | >200 | 59 | NA |
|  | M, 85 | Pfizer | 5 days after the first dose | IgG+, C3+ along the DEJ. | NA | NA | NA | NA |
|  | F, 83 | Moderna | 8 days after the first dose | neg | neg | neg | neg | NA |
|  | F, 66 | Pfizer | 7 days after the first dose | neg | neg | neg | neg | NA |
|  | F, 70 | Moderna | 9 days after the first dose | neg | NA | NA | NA | NA |
|  | F, 83 | Pfizer | 7 days after the second dose. | NA | NA | NA | NA | NA |
| Larson | M, 76 | Pfizer | 21 days after first dose | C3+/IgG+ along the DEJ. | IIF (SSS): roof | NA | NA | NA |
|  | M, 84 | Moderna | 14 days after second dose | C3+/IgG+ along the DEJ. | NA | NA | NA | NA |
| Gambichler | M, 80 | Pfizer | 7 days after the first dose | C3+/IgG+ along the DEJ. | IIF (SSS): roof | 365 | 223 | NA |
|  | M, 89 | Pfizer | 2 days after the first dose | C3+/IgG+ along the DEJ. | IIF (SSS): roof | 115 | 41 | NA |
| McMahon (12 cases) | sex: NS, age: 42-97 | Moderna (36%), Pfizer (64%) | N.A. | IgG+/C3+ (5/8), IgG+ (1/8) | anti-BP180: + (1/1) | NA | NA | NA |

NA=not available; DIF=direct immunofluorescence; IIF=indirect immunofluorescence; SSS=salt-split skin; ELISA=Enzyme-linked immunosorbent assay.
